# Influence of Talc Substitution with Starches from Different Botanical Origins on Rheological and Absorption Properties of Stiff Zinc Oxide Paste Formulations

**DOI:** 10.3390/pharmaceutics17050627

**Published:** 2025-05-08

**Authors:** Dragana Zaklan, Nikola Davidović, Jovana Milutinov, Dejan Ćirin, Veljko Krstonošić, Nebojša Pavlović

**Affiliations:** Department of Pharmacy, Faculty of Medicine, University of Novi Sad, 21000 Novi Sad, Serbia; dragana.zaklan@mf.uns.ac.rs (D.Z.); 201755@mf.uns.ac.rs (N.D.); jovana.milutinov@mf.uns.ac.rs (J.M.); dejan.cirin@mf.uns.ac.rs (D.Ć.); veljko.krstonosic@mf.uns.ac.rs (V.K.)

**Keywords:** semisolid, dosage form, excipient, rheology, suspension, tapioca

## Abstract

**Background:** Zinc oxide paste is traditionally compounded and applied in the therapy of various skin conditions. However, prolonged use of talc, usually present in zinc oxide pastes, may pose health risks due to potential contamination with asbestos and quartz, highlighting the need for alternative excipients. This study aimed to examine the effects of starches from various botanical sources and their particle size on the rheological and absorption properties of zinc oxide paste. **Methods:** Eight zinc oxide paste formulations were prepared, containing 25% zinc oxide and 25% indifferent excipient (talc, tapioca, rice, or maize starch) in two particle sizes. Rheological properties were assessed using amplitude and frequency sweep tests, and water- and oil-absorption capacities were determined using a centrifugation-based method. **Results:** Amplitude sweep tests confirmed the predominant solid-like nature of zinc oxide pastes, with the elastic modulus (G′) exceeding the viscous modulus (G″) in all formulations. Tapioca starch-based pastes exhibited the highest G′ and G″ values, while talc-based pastes exhibited the lowest. Frequency sweep tests showed that pastes were resistant to structural changes under stress, with G′ consistently dominating over G″ across the entire frequency range. Tapioca starch-based formulations exhibited the highest water-absorption capacity, while the talc-based formulations had the highest oil-absorption capacity. Reducing particle size improved both water- and oil-absorption capacities. **Conclusions:** Starches may be considered as alternatives to talc in zinc oxide pastes, due to their ability to modify the absorption and rheological properties of pastes. Future studies should assess the impact of starch substitution on sensory characteristics, shelf-life stability, and patient satisfaction.

## 1. Introduction

Topical therapy remains the most common treatment for a broad spectrum of cutaneous diseases. Semisolid pharmaceutical preparations are widely used in dermatology since they are easily administered, have cosmetic appeal, and provide drug retention at the application site. Furthermore, their physicochemical and sensory characteristics can be adjusted by selecting suitable pharmaceutical excipients and formulation strategies [1,2,3]. Pharmaceutical pastes are semisolid systems containing 20% to 50% adequately powdered insoluble components suspended in a hydrophobic or hydrophilic base. Pastes are commonly used for exudate binding, drying, and skin protection, particularly on areas with subacute or exudative lesions, due to their absorptive properties [3,4,5,6]. Despite their effectiveness, pastes are usually described as stiff, greasy, occlusive, and difficult to apply and remove. Thus, their practical and therapeutic relevance has lessened over time [4,5]. However, the use of pastes containing zinc oxide has been established over the years and recognized by worldwide expert authorities (e.g., U.S., German, British, and Balkan region countries’ pharmacopoeias and national formularies) [4,7,8,9,10].

Zinc has a long tradition of use as a topical agent and is referenced in the Ebers Papyrus and Ayurvedic literature [11]. Pharmacopoeias describe zinc oxide as a mild antiseptic, astringent, protective, and soothing agent. Zinc oxide is used extensively in pharmaceutical, cosmetic, and personal care products, and it is approved by the U.S. Food and Drug Administration (FDA) as generally recognized as safe [12]. Formulations containing this agent are of particular importance in the treatment of inflammatory skin conditions and wounds, such as venous and arterial leg ulcers, leprosy ulcers, burns and other mediums to heavy exudating healing wounds, warts, irritant diaper dermatitis, hand eczemas, as well as postoperative sun protection [6,13,14,15]. Namely, zinc displays anti-inflammatory, antibacterial, and antifungal effects; promotes epithelialization; and may initiate a cascade of immunological processes [11,14,15,16]. For instance, pastes containing zinc oxide combined with other active pharmaceutical ingredients and excipients are used to treat subacute and chronic psoriasis, chronic atopic eczema, and hyperkeratotic skin disorders [17].

Zinc oxide paste is one of the most common galenic pharmaceutical formulations. It usually consists of zinc oxide (25%) and talc (25%) suspended in white or yellow petroleum jelly (50%), making it an inexpensive and widely available product used for its wound-healing, skin-protective, exudate-absorptive, and drying properties [10,18]. Talc is a naturally occurring phyllosilicate mineral broadly applied in the cosmetic and pharmaceutical industries. Its favorable physicochemical properties encompass its brightness, particle shape, softness, smoothness, and inertness. Furthermore, it has very good oil and grease absorption capacity, prevents caking, and improves the flowability and compressibility of powders. Thus, it is commonly utilized in the formulation of solid and semisolid pharmaceutical dosage forms and cosmetic products [19,20,21]. However, the use of talc has been under scrutiny and substantially decreased over the last decades. In particular, the softest known mineral often co-occurs with asbestos and quartz, both class 1 carcinogens according to the International Agency for Research on Cancer (IARC), which also states that asbestos-contaminated talc should be referred to as carcinogenic [22,23]. Asbestos exposure is linked to various morbidities, including asbestosis, mesothelioma, and lung and ovarian cancer [24,25]. The controversy has been ongoing since the 1970s, and the U.S. FDA is continuously testing talc-containing cosmetics for asbestos [26,27]. Occasionally, contaminated cosmetic and personal care products still reach consumers [25]. The American Cancer Society stated that people concerned about using talc-containing products may want to avoid them until more information on the safety is made available [28]. For these reasons, the search for safer pharmaceutical and cosmetic ingredients is of major significance.

Starch is a semi-crystalline biopolymer composed of linear amylose and branched amylopectin molecules and is a major polysaccharide in plants. Being a major macro-component in many foods, its significance and use in human nutrition has long been established. The global market for starch was estimated at 134.5 million metric tons in 2022, produced for both edible and non-edible purposes, due to its versatile functionality [29,30]. Starches of various botanical origins differ in their characteristics. However, the physicochemical, morphological, thermal, and rheological properties can be altered, provided they undergo chemical modification [31,32]. Both native and modified starches have long been utilized as pharmaceutical excipients in conventional and advanced drug delivery. Not only are they characterized by favorable functionality, but they are also versatile, inexpensive, biodegradable polymer materials available from renewable sources, which makes them attractive to the various industries, including pharmaceutical, commonly serving as binders, disintegrants, diluents, glidants, and lubricants in the formulation of solid and semisolid dosage forms [33]. In this sense, starch has been used as a talc replacement, and some of the authorities list it as the excipient in zinc oxide paste [7,8,10].

As previously mentioned, topical medicines are widely used in the treatment of skin diseases. Nevertheless, relatively low adherence, poor cosmetic acceptability, and low satisfaction with topical treatments have been reported. Patient preferences in terms of the rheological properties of semisolid dosage forms need to be particularly considered in the process of product development, as they significantly influence adherence and clinical outcomes, and a change of the excipient is known to affect the rheological profile of a formulation [3,34]. Given the potential of starches as natural, safe alternatives to talc, we hypothesized that starches from different botanical origins and particle sizes would significantly affect the rheological and water- and oil-absorption properties of zinc oxide pastes. Therefore, this study aimed to systematically evaluate these effects and identify the most promising starch type for talc substitution.

## 2. Materials and Methods

### 2.1. Materials

**Figure 1:**
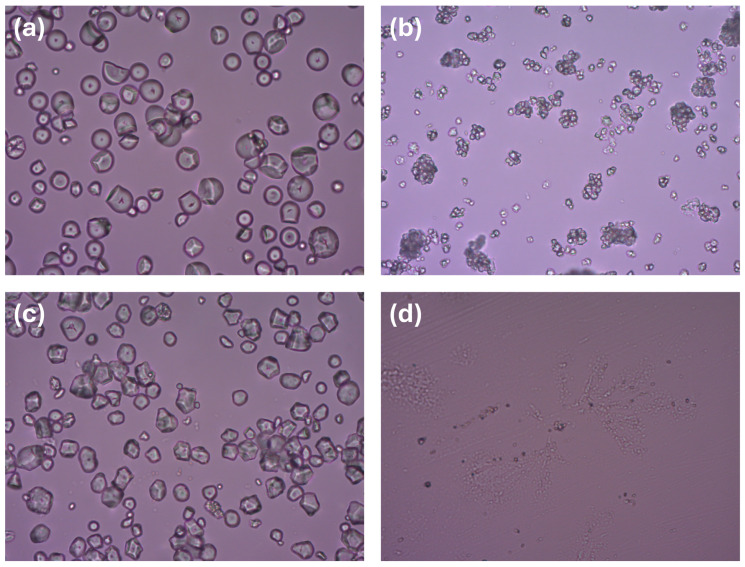
Microphotographs of starch granules and talc under normal light (magnification 400×): (**a**) tapioca starch, (**b**) rice starch, (**c**) maize starch, (**d**) talc.

For the paste preparation, the following substances were used: zinc oxide (Lot No.: 54023, Norzinco GmbH Harzer Zincoxide, Bad Harzburg, Germany), tapioca starch (Lot No.:110924, Bio Špajz d.o.o., Beograd, Serbia), rice starch (Bach No.: 2017-1295, Lach: ner, Neratovice, Czech Republic), maize starch (Lot No.: L1131234/239, Jabuka, Pančevo, Serbia), talc (Lot No.: 19000209/02018067G, MAGNESIA GmbH, Lüneburg, Germany), and white soft paraffin (Lot No.: 0000393594, AlekPharm, Beograd, Serbia). All excipients were microscopically characterized first (Figure 1). For sorption tests, olive oil (Lot No.: 0011123, AlekPharm, Beograd, Serbia), paraffin oil (Lot No.: 2189/08/20, Centrohem, Stara Pazova, Serbia), and purified (distilled) water were used.

### 2.2. Zinc Oxide Paste Formulation Design and Preparation Method

Eight stiff zinc oxide paste formulations were prepared using four different excipients (tapioca starch, rice starch, maize starch, and talc), each in two particle sizes (140 μM and 355 μM) obtained by sieving (Retsch AS 200, Haan, Germany). Pastes were prepared by adding the melted white soft paraffin in portions to the previously prepared mixtures of zinc oxide and indifferent excipients having specified particle sizes. Eventually, the entire mixture was homogenized thoroughly for 20 min, following the principles of magistral compounding, using pestle and mortar. The quantitative composition of prepared pastes is shown in Table 1.

Pastes were evaluated for their physical appearance. They were set in Petri dishes and inspected visually for their color, homogeneity, and consistency. For homogeneity testing by visual inspection, pastes were set between two glass plates in order to observe the potential presence of lumps and aggregates.

### 2.3. Rheological Studies

Rheological properties of pastes were determined 24 h after their preparation by Haake Mars rheometer (Thermo Scientific, Bremen, Germany) using a plate–plate geometry (P35/Ti) at 25 ± 0.1 °C. First, the pastes were exposed to the increasing shear rates; however, it was not possible to perform continuous flow (hysteresis loop) tests due to the occurrence of the rod-climbing or “Weissenberg effect”, which caused material climbing behavior under shear, indicating strong elasticity. Oscillatory experiments were performed through amplitude and frequency sweep tests. Amplitude sweep tests were used to determine the linear viscoelastic region (LVR). Elastic modulus G′ and viscous modulus G″ were recorded versus shear stress (1–1000 Pa) at constant frequency of 1 Hz. The tangent of the phase angle (tan δ = G″/G′), a dimensionless and a relative measure of the viscous and elastic properties of a material, was also calculated. The plateau values of G′ indicating the structural integrity of the material within the LVR were determined, as well as the end of the LVR as a decrease in G′ plateau values for more than 10%. Yield stress (τ_0_), i.e., the point where the material begins to flow or undergo significant structural rearrangement, was calculated from amplitude sweep test results as the shear stress at which tan δ deviated by 10% from its initial plateau value, marking the end of the LVR. The appropriate strain, selected as the middle of the LVR, was applied in frequency sweep tests for the pastes. In frequency sweep experiments, G′ and G″ moduli were recorded versus frequency (0.1–10 Hz) at constant shear stress of 50 Pa that was selected from the amplitude sweep test results.

### 2.4. Sorption Properties Measurement

Sorption capacities of zinc oxide pastes towards water, plant oil (olive oil), and mineral oil (paraffin oil) were determined following the method described by Benavent-Gil and Rosell [35] with slight modifications. Samples (0.50 g ± 0.01 g) were mixed on centrifuge tubes with 5 mL of distilled water or oils and centrifuged at 3500 rpm for 10 min (Sigma Laborzentrifugen, Osterode am Harz, Germany). Each measurement was performed in triplicate. Absorption capacities (%) of pastes towards water and oils were expressed as a percentage weight of solvent retained by the sample, calculated as:

(1) $$\mathrm{Absorption}\;\mathrm{capacity}\;(\%)=\frac{Sample\;mass\;after\;centrifugation\;\left(g\right)-Initial\;sample\;mass\;(g)}{Solvent\;mass\;(g)}\times 100$$

### 2.5. Statistical Analysis

All data are presented as a mean ± standard deviation (SD). For multiple group comparisons, one-way analysis of variance (ANOVA) followed by Tukey’s post hoc test at *p* < 0.05 was applied using OriginPro 2019 software (Originlab, Northampton, MA, USA).

## 3. Results

### 3.1. Visual Inspection of Zinc Oxide Pastes

The organoleptic evaluation showed that all the tested paste formulations were white in color with a very thick consistency, rough but generally homogeneous texture, and some unfavorable tactile properties, such as poor skin spreadability. No larger solid particle aggregates were observed after placing the samples between two glass plates, indicating good homogeneity of all formulations.

### 3.2. Rheological Properties of Zinc Oxide Pastes

**Figure 2:**
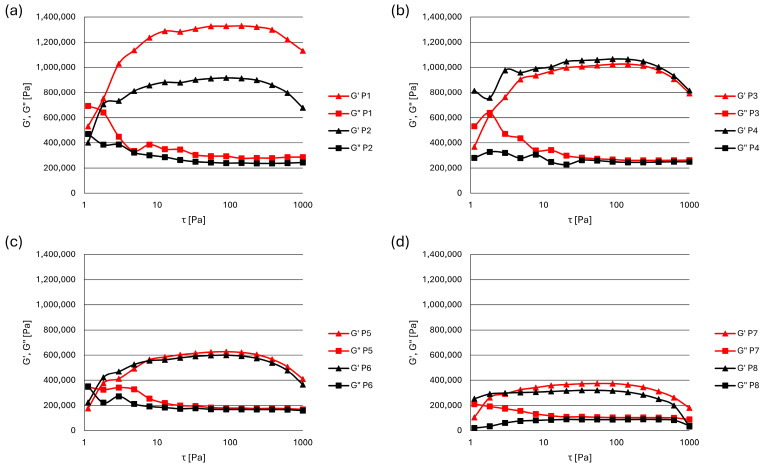
The changes in elastic (G′) and viscous (G″) moduli with shear stress at a constant frequency in zinc oxide pastes with: (**a**) tapioca starch, (**b**) rice starch, (**c**) maize starch, (**d**) talc.

Dynamic oscillatory shear tests were performed by subjecting the zinc oxide pastes to a sinusoidal deformation and measuring the resulting mechanical response. First, amplitude sweep tests were used for determining the linear viscoelastic region (LVR), when the frequency was fixed, and the applied stress amplitude varied from 1 to 1000 Pa. The effects of the stress amplitude on the dynamic properties of all paste formulations are shown in Figure 2.

Very high values of both the elastic (G′) and viscous modulus (G″) were recorded for all formulations. The elastic modulus reached values of above 1 MPa in pastes with tapioca starch (P1) and rice starch (P3, P4). For all samples, several times higher values of G′ compared to G″ were measured, which indicates that the elastic (solid-like) properties dominate over the viscous (liquid-like) properties in the formulated stiff zinc oxide pastes. Accordingly, the values of the tangent of the phase angle (tan δ) defined as the ratio of G″ to G′ remained below one for all pastes, confirming their predominantly elastic behavior, which correlates with better structural stability. These values were mostly stable in the whole range of the applied shear stress, and they were in the range of 0.2221–0.2946 at 50 Pa, as the middle of the LVR (Table 2). Besides having the highest values of G′ and G″, the pastes with tapioca starch were also characterized by the influence of particle size on the rheological properties, which was more pronounced in comparison to that in other paste formulations. In pastes with tapioca starch, higher values of G″ and especially G′ were observed in the formulation with smaller starch particles (P1 vs. P2). Accordingly, when comparing the tan δ values of pastes containing the same excipients in different particle sizes, a statistically significant difference was observed only for the tapioca starch-based pastes (P1 vs. P2, *p* < 0.05). The lowest values of G′ and G″ were recorded for stiff zinc pastes with talc (P7, P8). In all the tested formulations, the existence of LVR was determined to be in the range of shear stress from 10 to 100 Pa.

The plateau values of G′; the end of the G′ plateau, i.e., the shear stress values at which G′ decreases by more than 10%; tan δ values at 50 Pa (middle of LVR); and the yield stress (τ_0_) as the minimum stress required to initiate flow calculated from tan δ values are presented in Table 2. The plateau values of G′ indicate the structural integrity of pastes in their LVR, while the end of the LVR may be expressed as the shear stress at which G′ starts to decrease or tan δ starts to increase. G′ plateau values ranged from 3.146 × 105 Pa (P8) to 13.06 × 105 Pa (P1), and accordingly, the shear stress values at which the G′ plateau (LVR) ended were ranged from 237.7 Pa (P8) to 846.6 Pa (P1). In all formulations, the pastes with smaller particles had a broader LVR (the end of the LVR at higher shear stresses). Both higher G′ plateau values and shear stress values of the end of G′-based LVR indicate a stronger internal network. The G′-based and tan δ-based LVR limits differed, indicating variations in the structural response of pastes depending on the chosen parameter for defining the end of the LVR; however, a similar trend was observed. Since the values of G″ decreased proportionally along with G′, the end of the tan δ-based LVR (yield stress, τ_0_) occurred at higher shear stress values, with four formulations having τ_0_ around or above 1000 Pa. The lowest τ_0_ values, significantly different from all other formulations, were observed for talc-based zinc oxide pastes (237.7 Pa for P8 and 292.5 Pa for P7).

**Figure 3:**
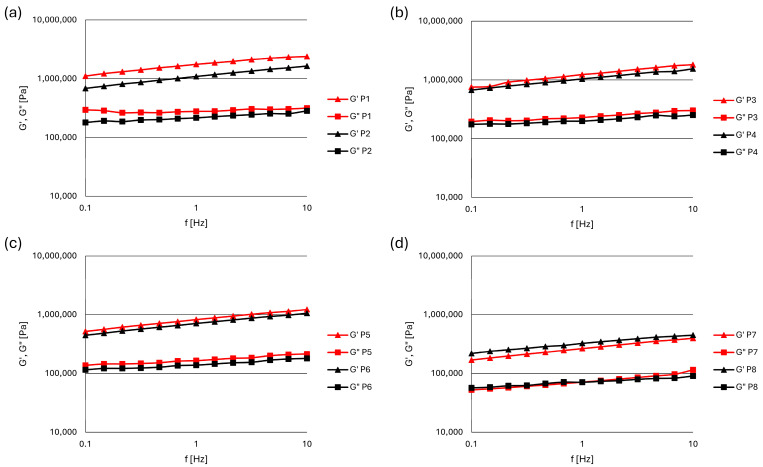
The changes in elastic (G′) and viscous (G″) moduli with frequency at a constant shear stress in zinc oxide pastes with: (**a**) tapioca starch, (**b**) rice starch, (**c**) maize starch, (**d**) talc.

In another oscillatory test (frequency sweep test), the time-dependent behavior of pastes was measured by varying the frequency of the applied stress within the LVR, i.e., in the non-destructive deformation range. The effects of increasing the frequency of deformation from 0.1 to 10 Hz on the dynamic properties of zinc oxide paste formulations within the LVR (at 50 Pa) are shown in Figure 3. Higher values of the elastic (G′) modulus than the viscous (G″) modulus and a parallel increase in both moduli were recorded for all pastes, which indicates that they exhibit viscoelastic, dominant elastic (solid-like) behavior across the whole tested frequency range. The values of G′ and G″ were the lowest in pastes with talc (P7, P8) and the largest in the formulations with tapioca and rice starch (P1, P2, P3, P4). The influence of particle size on the dynamic properties of pastes in the frequency sweep test was significant only in pastes with tapioca starch (Figure 3a).

**Figure 4:**
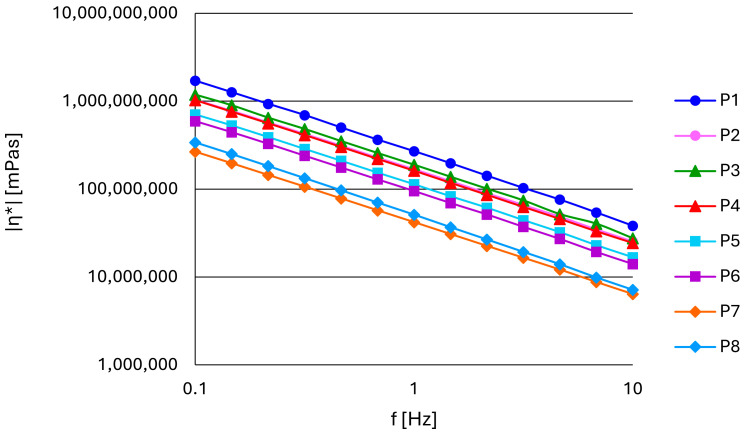
The change in complex viscosity (η*) with frequency at a constant shear stress in zinc oxide pastes.

The complex viscosity (η*), as the viscosity measured in oscillatory rheological experiments, represents the total resistance to flow as a function of angular frequency. The increase in stress frequency led to the reduction in complex viscosities in all zinc oxide pastes (Figure 4), which suggests that the structural dynamics of the material allow for easier deformation at higher deformation rates. The pastes with talc (P7, P8) had significantly lower values of complex viscosity in comparison to the paste formulations with starches. No significant differences in the complex viscosity values were observed, when comparing pastes with the same excipient of different particle sizes.

### 3.3. Sorption Properties of Zinc Oxide Pastes

**Figure 5:**
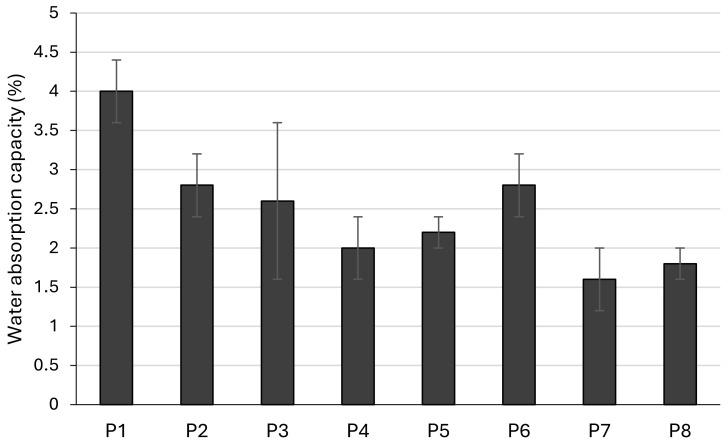
Water absorption capacity (%) of zinc oxide pastes.

Water absorption capacities (WACs) of the tested zinc oxide pastes ranged from 1.6 to 4% (Figure 5). The highest values of this parameter were obtained for pastes with tapioca starch (P1, P2) and the lowest for pastes with talc (P7, P8). The influence of the particle size was statistically significant only in the case of pastes with tapioca starch (P1 vs. P2). The paste with a smaller particle size of tapioca starch (P1) exerted significantly better sorption characteristics towards water than all other zinc oxide paste formulations (*p* < 0.05).

**Figure 6:**
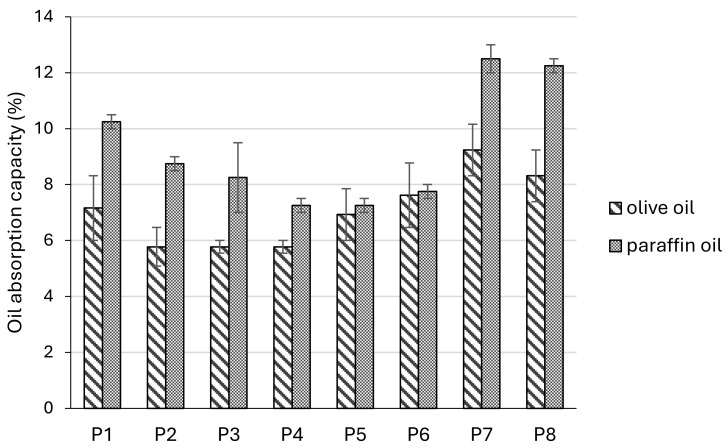
Oil absorption capacity (%) of zinc oxide pastes towards olive oil and paraffin oil.

Oil-absorption capacities (OACs) of zinc oxide pastes were determined using a plant (olive) and a mineral (paraffin) oil. The obtained absorption capacities were higher in comparison to the water absorption values. Moreover, OAC values were higher when paraffin oil was used in comparison to olive oil, and they were over 12% for pastes with talc towards paraffin oil (Figure 6). The pastes with talc (P7, P8) had significantly higher OACs towards olive oil than pastes with tapioca and rice starch (P2, P3, P4) and significantly higher OACs towards paraffin oil than all pastes with starches (P1–P6). Smaller particle sizes generally led to an increase in the OACs towards both types of oil; however, it was statistically significant only in the case of pastes with tapioca starch towards paraffin oil (P1 vs. P2).

## 4. Discussion

Topical drug formulations are widely used for the local treatment of various skin disorders but also applied for the purposes of systemic drug delivery. Hence, the search for innovative, safe, and efficacious topical therapeutic modalities is of the utmost importance. In addition, the aesthetic and sensory characteristics of such products need to be properly addressed in order to meet patients’ demands in terms of cosmetic appeal and ultimately improve adherence and therapeutic outcomes. In addition, the growing demand for sustainability in pharmacies and products based on natural ingredients should not be overlooked [34,36].

The sensory analysis has been extensively used to evaluate the cosmetic and organoleptic characteristics of topical products. The results are usually reliable and consistent; however, as it requires a group of well-trained panelists, it is both time-consuming and costly, and the subjectivity cannot be completely ruled out [37]. Thus, laboratory methods and instrumental measurements have been introduced with the aim of obtaining more objective information and reducing analysis time and costs. In this sense, rheology is one of the most studied and utilized methods, since rheological parameters have proven to correlate well with sensory attributes provided by human panelists [37]. Most of all, product flow behavior and various textural characteristics can be assessed by employing rheological measurements [38]. Flow properties of the final product significantly affect the application and acceptance of the semisolid dosage forms, and the yield stress and dynamic viscosity correlate with primary skin feeling [38,39].

In this sense, it is expected that the change in the formulation of pharmaceuticals can significantly affect the sensory attributes and, consequently, patients’ acceptance of the product. Therefore, we aimed to investigate the effect that various excipients and their particle sizes have on the rheological and absorption properties of stiff zinc oxide pastes. A summary of the key findings of the study is provided in the Supplementary Materials. First, the rheological properties of pastes were determined by measuring the deformation behavior of the samples. Rheological properties can be divided into viscous, elastic, and plastic [40]. Viscoelasticity encompasses both viscous and elastic properties and is characteristic of semisolid systems. The most widely used technique for determination of microstructural characteristics and viscoelastic properties of a material is dynamic rheology, performed under oscillatory shear [41]. The amplitude sweep test measures the degree of linearity of the formulation and is the first step in evaluating the viscoelastic character of semisolids. The LVR is the region in which a sample maintains its structure when exposed to increasing stress at a constant frequency, providing information on its structure and firmness. The longer the LVR, the more structured and firmer the sample is, while the shorter LVR indicates less firm material [40,42]. Furthermore, the frequency sweep test provides the insight into the structural profile of the sample by exposing it to a stepwise increase in frequency and a constant sinusoidally varying deformation stress or strain. The elastic/storage modulus (G′) is a measure of energy stored elastically during a cycle of oscillation and presents the solid-like nature of the sample, whereas viscous/loss (G″) is a measure of energy dissipated at viscous flow during oscillation and mirrors the liquid-like quality of the sample. The loss tangent (tan δ) is another rheological parameter associated with the energy dissipated per cycle divided by the energy stored per cycle (G″/G′). Thus, its values indicate whether the behavior of the studied material is elastic (tan δ < 1) or viscous (tan δ > 1) [36,40,43].

The amplitude sweep test was used to investigate the viscoelastic properties of various zinc oxide pastes, and the results showed that the LVR existed in the range of 10 to 100 Pa for all tested samples. Furthermore, G′ values were dominant over G″ in all the tested formulations, suggesting that the elastic component was more pronounced than the viscous one, indicating a certain stiffness or rigidity in the sample structure. Since no crossover of the elastic and viscous moduli was observed, the pastes are expected to be non-sticky [41,42]. The highest values of G′ and G″ were obtained for the tapioca starch pastes. Moreover, the influence of the particle size was the most pronounced in this specific system, as the decrease in particle size caused an increase in the paste stiffness (P1 vs. P2). Thus, tapioca starch-containing pastes are expected to have the highest ability to maintain their shape or structure when exposed to force [42]. Conversely, we observed that in the case of rice starch paste, larger particles produced a firmer sample, although this effect was not as pronounced as that in tapioca paste (P4 vs. P3). The lowest values for both G′ and G″ were observed in the zinc oxide paste formulated with talc. The substitution of talc with starches of different botanic origins in the zinc oxide paste composition caused more prominent alterations in the elastic behavior than in the viscous behavior of the samples. The values of tan δ were similar in all systems and additionally confirmed the existence of the LVR ranging from 10 to 100 Pa, as well as the prevailing solid-like character of the samples. Yield stress (τ_0_), calculated from tan δ values, represents the minimum stress required to initiate deformation or flow in a material, and it was shown that talc-based zinc oxide pastes, particularly the one with the larger particle size (P8), were the least resistant to flow and significantly different from starch-based pastes. As the spreadability of the topical formulation appears to be negatively associated with the yield stress, talc-based pastes are expected to be easier to spread on skin [36].

The results of the frequency sweep test provide information on the inner structure of the material. Pastes formulated with tapioca and rice starch displayed the highest values of the measured parameters under the increasing frequencies. A parallel increase in G′ and G″ in all pastes suggests that the relative balance between elastic and viscous contributions remains constant over the frequency range. This often occurs when the structure or interactions within the material provide proportional resistance to deformation and energy dissipation. The inner structure of pastes remains intact and behaves predominantly like a solid throughout the frequency sweep range, indicating their resistance to structural changes under stress [40].

Although it was not possible to perform continuous flow tests due to the occurrence of the Weissenberg effect, complex viscosities were calculated from oscillatory measurements. The Cox–Merz rule is an empirical relationship demonstrating that the steady shear viscosity plotted against shear rate is correlated with the complex viscosity plotted against frequency [44]. Therefore, the decrease in complex viscosities with an increase in frequency indicates a shear-thinning rheological behavior of all tested pastes. Complex viscosities measured within the LVR were similar for pastes containing starch of different botanical origins (P1–P6). However, pastes containing talc were characterized by an evidently lower complex viscosity. Again, the particle size did not have a distinct impact on the values of the aforementioned parameter.

The characterization of structured materials such as starch and investigation of their viscoelastic properties are of great value when attempting to define their functionality. Starch has long been in the focus of food science and industry, especially applied as a modifier of rheological and textural properties of foodstuff, stabilizing, gelling, and binding agents [45]. The polymerization and structuration of starch are controlled by genetic mechanisms, environmental factors, and growing conditions [46]. Furthermore, starch granule size and its fine structure, amylose/amylopectin ratio, and molecular weight majorly affect the functional properties of this biopolymer, including gelatinization and pasting properties, crystallinity, swelling, and solubility [47]. Research in this field has demonstrated that various starches may have chemical similarities but fundamentally different functional properties. The content of amylose, a linear chain of D-glucose linked by α-1,4-glycosidic bonds, is recognized as a factor that impacts starch’s functional properties to a significant extent. However, they can also be altered by fine-tuned variations in the structure of amylopectin, which is a highly branched polymer composed of D-glucose units linked by α-1,4-glycosidic bonds, with α-1,6 branching links [30]. Normal starches consist of 20–30% amylose and 70–80% amylopectin, although this ratio may vary naturally or be physically or chemically modified. Previous studies have reported that starches with higher content of amylose had higher G′ and G″ and lower tan δ [48]. Amylose and amylopectin are stored in the form of starch granules that are characterized by a complex structure. According to their dimension, starch granules are classified as A- (>15 μm), B- (5–15 μM), or C-type (<5 μM), and their size, shape, and proportion depend on the botanical source [31]. Granule size and shape significantly affect texture, consistency, and other attributes of both food and non-food starch items. Furthermore, the particle size of starch powder reflects on other aspects of starch utility, including handling, packing, and product formulation [49]. In this study, we used tapioca starch that is characterized by high content of spherically shaped B-type granules and up to 25% amylose content, rice starch with small granules of polyhedral shape and up to 30% amylose, and maize starch with spherical and polygonal granules with up to 40% amylose [31,50,51,52].

Mauro et al. investigated the impact of starch concentration on the rheological properties of gluten-free gels. The results obtained from the frequency sweep test showed that all samples, including those containing tapioca, normal rice, or normal maize starch, had higher values of the elastic modulus than the viscous modulus, and consequently, tan δ < 1. Furthermore, the authors showed that the value of the elastic modulus increased with the starch concentration in each case studied, although the nature of the starch influenced its evolution. Elastic properties were most pronounced in the gels made from normal corn starch, followed by those of normal and waxy rice starch. Tapioca gels had lower elasticity and displayed higher loss tangent values, which increased with concentration [51]. The results of our study are in accordance with the aforementioned, as all pastes containing starch as the excipient showed distinctive elastic behavior. However, the zinc oxide paste containing tapioca starch was identified as the one with the highest G′ and G″ values. Sun and Yoo explored the rheological properties of different rice starch and tapioca starch blends. In this study, both the G′ and G″ values of rice starch paste were higher than those in tapioca starch paste. Furthermore, the dynamic moduli values of rice starch paste decreased after adding tapioca starch and were influenced by their mixing ratio. The changes in G′ values were greater than the changes in G″, indicating that the elastic properties of rice starch paste were more affected by the addition of tapioca starch in the blend [53]. Nevertheless, our research revealed that tapioca starch as the excipient produced zinc oxide pastes with the highest G′ values, as determined in both the amplitude sweep and frequency sweep tests. Although the particle size evidently influenced the values of the elastic and viscous moduli in the amplitude sweep test, that was not the case in the frequency sweep test performed in the LVR. Furthermore, when compared with conventional zinc oxide/talc formulation, both tapioca and rice starch pastes had significantly higher values of G′ and G″ in the samples compounded with particles larger in size. On the other hand, this was not the case with maize starch pastes, as they were the most similar to the conventional formulation. Bearing in mind that the compliance for pharmaceutical or market success of cosmetic semisolids does not depend only on their efficacy and safety but also on aesthetic and organoleptic characteristics, assessment of sensory characteristics at an early stage of product development is of vital importance [36,42]. For instance, when investigating the relationship between the sensorial and physical characteristics of topical creams, Ali et al. revealed that modified quinoa starch surfactant-free Pickering oil-in-water cream with carbomer was characterized by the human panel as preferable in the after-feel phase of the study. This cream was perceived as the one with the least stickiness, greasiness, residual coating, and slipperiness but was also the least soft [34]. That being said, it is clear that the influence of starches in the formulation of stiff zinc pastes is also reflected in their sensory characteristics, which can positively or negatively affect the acceptance of these topical formulations by patients. Subsequently, these sensory modifications may enhance generally low patient adherence, especially in pediatric or geriatric populations, where product acceptability is critical [3].

Due to the high proportion of fine powders they contain, stiff pastes can absorb exudates and thus have drying properties [4]. In this study, we investigated water- and oil-absorption capacities of the compounded zinc oxide pastes. Water absorption capacities (WAC) ranged between 1.6% and 4%. The highest values were obtained for pastes containing tapioca starch (P1 and P2) and the lowest for conventional formulations with talc (P7 and P8). Furthermore, the influence of the particle size was most pronounced in tapioca pastes, as the formulation with smaller particles had significantly higher WAC compared to its counterpart. When comparing formulations prepared with a particle size of 140 μm, WAC had the following rank order: tapioca starch > rice starch > maize starch > talc. On the other hand, formulations prepared with particle size of 355 μm had the following WAC rank order: maize starch > tapioca starch > rice starch > talc. Higher WAC, particularly in tapioca-based pastes, suggests enhanced performance in absorbing wound exudates, making them suitable for weeping skin conditions [54]. The capacities of zinc oxide pastes to absorb plant oil and mineral oil (OAC) were also evaluated. Evidently, the tested samples exerted higher OACs than WACs. Furthermore, the olive oil absorption capacity ranged between 5.8% and 9.2%, while paraffin oil absorption capacity ranged between 7.2% and 12.2%. Overall, talc pastes absorbed the highest amounts of oils, while the decrease in particle size increased the OAC of tapioca starch, rice starch, and talc formulations. Olive oil OAC had the following rank order: talc > maize starch > tapioca starch > rice starch. On the other hand, paraffin oil OAC ranked as: talc > tapioca starch > rice starch > maize starch. In summary, zinc oxide paste compounded with tapioca starch had the best WAC, and the conventional formulation with talc possessed the highest OAC, while the reduction in particle size favorably affected both WAC and OAC. Juch et al. reported no water absorption properties for the lipophilic wheat starch-containing zinc oxide paste, either before or after pre-drying, suggesting that the components which form the vehicle of the paste significantly impact its water uptake capacity [4]. Aforementioned research by Mauro et al. reported that the lowest values for WAC among tested starch samples were obtained for normal rice and tapioca starch and were higher for normal maize. However, they did not detect the substantial difference in WAC or other investigated parameters, including swelling power and water solubility index, stemming from the variability of amylose content among tested starch samples. However, a study by Bhat and Riar reported a positive correlation of starch WAC with amylose content [49]. Other factors apart from amylose content determine the functional properties of starches, including the structure of amylopectin and the interactions between amorphous and crystalline zones [51]. The molecular size and chain distribution of amylopectin influence the formation of hydrogen bonds [55]. Furthermore, the reduction in amorphous regions in starch granules may be one of the reasons for its lower water binding capacity [56]. In that sense, starch granule type also significantly impacts starch–water interactions. X-ray diffraction results showed that A-type granules are slightly more crystalline than B-type granules, and water molecules are less free to migrate into A-type granule starches. B-type granules, on the other hand, are less orderly arranged, meaning they possess higher affinity towards water and are more hydrated at room temperature. Higher WACs of B-type granules may also be due to their greater surface area, compared to their A-type counterparts [57]. In summary, the WAC of starches significantly depends on their botanical source [35]. The amylose/amylopectin ratio, chain length, and molecular weight distribution and the degree of branching determine the hydration capacity of starches, as well [58]. The variations in obtained WAC values could be attributed to the variations in starch structure, which affect its ability to form covalent and hydrogen bonds between the chains and ensure the availability of water binding sites [51]. The ability of starches to bind lipids is also dependent on the amylose content, as this biopolymer is the one that mainly forms inclusion complexes with fatty substrates. Previous analysis showed that aliphatic moieties of lipids may be inserted at the ends of amylose chains and included inside the amylose helix, while the polar domains of the lipids are too bulky to enter it. On the other hand, a high degree of branching limits the lipid binding capacity of amylopectin [30]. The WAC and OAC in various rice starches were reported to be in accordance with their particle size, as bigger particles possessed higher WACs and OACs. Moreover, the morphology of the starch particles influences the values of the mentioned parameters. For instance, starches with spherical morphology were found to have lower WACs and OACs than those with polyhedral morphology, most likely due to the smaller surface area of spherical particles [49]. The results of our study show that spherically shaped tapioca starch exerted the highest WAC, especially in the formulation compounded with particles smaller in size. Moreover, all pastes formulated with starches had higher WACs than that of the conventional formulation of zinc oxide paste with talc. On the other hand, the substitution of talc with each of the starches led to a decrease in OAC values. The absorbing capacity of talc is well known in the literature, and it is widely used as a cosmetic ingredient for its water and oil binding properties, among others. Talc’s surface can strongly absorb individual water molecules and exhibits a hydrophilic nature, but as water droplets saturate its surface, water–water interactions overtake, and cohesion becomes stronger than adhesion between the talc surface and water [59,60]. Regarding the implications of these findings on sensory properties, the overall benefit of using starch in zinc oxide pastes could be related to the after-feel. Desirable sensations such as dry and powdery after-feel may replace generally not preferable sensations of stickiness and greasiness [34].

Regarding the constituents of the compounded pastes, stability considerations should be made, since they directly impact the quality of the final preparation. Both microbiological stability and quality of compounded medicines are essential to ensure their safety and efficacy. These factors are extensively addressed in various pharmacopoeias, national formularies, and regulatory guidelines. The potential microbial contamination of the raw materials should be of special concern [61,62]. Starch is generally stable in storage for a prolonged period when kept under dry conditions. However, exposure to a hot and humid environment may considerably increase its susceptibility to microbial contamination [33]. Thus, to minimize the risk of contamination of compounded dermatological formulations, it is imperative that adequate precautionary measures are taken during starch handling and storage. Although the lipophilic nature of the compounded zinc oxide paste contributes to formulation stability so that the expected beyond-use date (BUD) ranges between six months and one year, conducting rigorous stability and microbiological testing is needed to secure formulation quality and safety [8,10,63]. In addition to a lack of textural and sensory analysis, a notable limitation of this study is the absence of microbiological stability testing, both for raw excipients and the final preparations.

## 5. Conclusions

Talc substitution with starches from different botanical origins significantly modifies the absorption and rheological properties of stiff zinc oxide paste formulations for topical use. Tapioca starch had the most pronounced influence on the rheological characteristics of the paste, especially on the elastic modulus. While particle size did not universally affect rheological behavior, it significantly influenced the absorption capacity in tapioca starch-based pastes. Pastes with all tested starches showed a more pronounced capacity for water absorption, along with lower oil absorption, compared to the conventional formulation with talc.

Therefore, starches hold significant potential as excipients in pharmaceutical paste formulations, particularly for managing moist skin conditions, where their water-absorptive capacity can play a crucial role. By varying the components of the formulation, rheological as well as water and oil binding capacities of conventional zinc oxide stiff paste may be optimized, with the aim of improving its sensory characteristics and, consequently, patients’ acceptance of the product and therapeutic outcomes. Our study finds that the substitution of talc with starches is justified in this context; however, it is necessary to perform further textural measurements and sensory analyses, as well as stability testing of both the raw material and final preparations, to exploit the full potential of starches as excipients in paste formulations for topical application.

## Figures and Tables

**Table 1:** Quantitative composition of zinc oxide pastes (% *wt*/*wt*).

	Particle Size	P1	P2	P3	P4	P5	P6	P7	P8
Zinc oxide	140 μM	25	25	25	25	25	25	25	25
Tapioca starch	140 μM	25	-						
	355 μM	-	25						
Rice starch	140 μM			25	-				
	355 μM			-	25				
Maize starch	140 μM					25	-		
	355 μM					-	25		
Talc	140 μM							25	
	355 μM								25
Soft paraffin	-	50	50	50	50	50	50	50	50

**Table 2:** Tangent of the phase angle (tan δ), plateau value of G′, the ends of G′-based and tan δ-based linear viscoelastic region (LVR) of zinc oxide pastes.

Formulation	tan δ * (G″/G′)	G′ Plateau [×10^5^ Pa]	End of G′-Based LVR [Pa]	End of tan δ-Based LVR, τ_0_ [Pa]
P1	0.2221	13.060	846.6	736.7
P2	0.2681	8.954	578.8	999.1
P3	0.2694	10.030	630.9	>1000
P4	0.2450	10.430	592.4	>1000
P5	0.2946	6.109	445.2	630.5
P6	0.2848	5.831	424.7	999.1
P7	0.2827	3.676	292.5	331.6
P8	0.2723	3.146	237.7	208.6

* at 50 Pa (middle of LVR)

## Data Availability

The raw data supporting the conclusions of this article will be made available by the authors on request.

## Supplementary Materials

The following supporting information can be downloaded at: https://www.mdpi.com/article/10.3390/pharmaceutics17050627/s1, Figure S1. Radar chart summarizing the rheological (G′ plateau) and absorption (water, paraffin oil, and olive oil absorption capacities) properties of stiff zinc oxide pastes.
