# Supplementary material for: Influence of Talc Substitution with Starches from Different Botanical Origins on Rheological and Absorption Properties of Stiff Zinc Oxide Paste Formulations
Source: Pharmaceutics. 2025 May 8;17(5):627. doi: 10.3390/pharmaceutics17050627 (PMC12115069; doi:10.3390/pharmaceutics17050627)
Supplement: Supplementary file 1 [file pharmaceutics-17-00627-s001.zip › pharmaceutics-3603035-supplementary.pdf]

Supplementary material

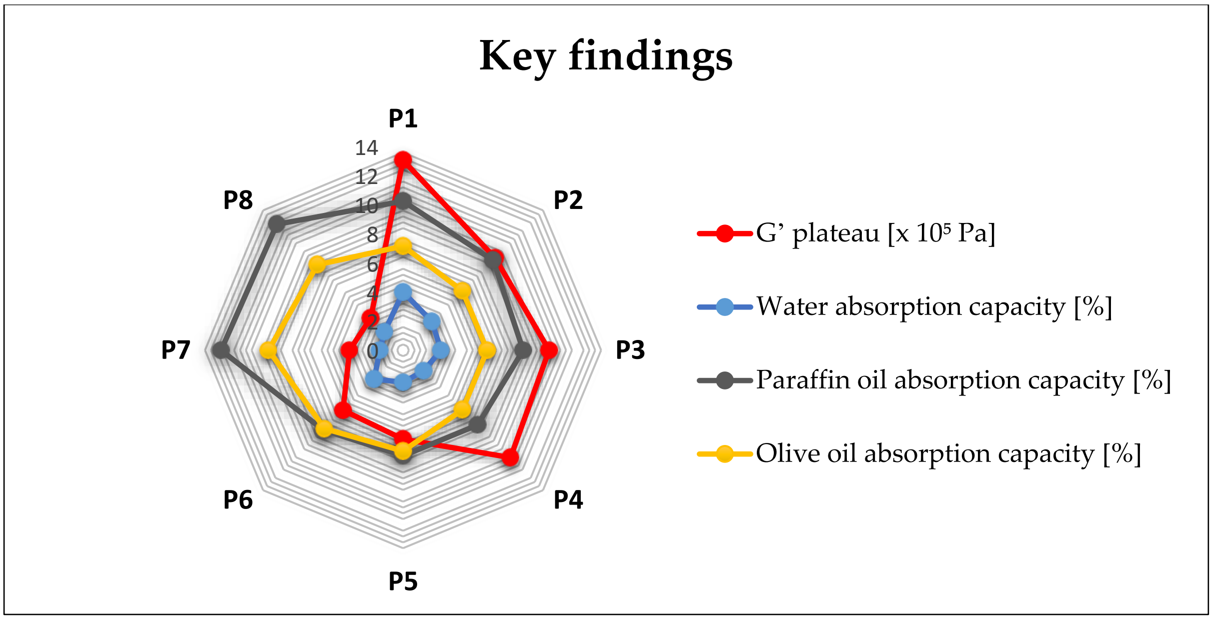

Figure S1. Radar chart summarizing the rheological (G' plateau) and absorption (water, paraffin oil, and olive oil absorption capacities) properties of stiff zinc oxide pastes.
